# The skin function: a factor of anti-metabolic syndrome

**DOI:** 10.1186/1758-5996-4-15

**Published:** 2012-04-26

**Authors:** Shi-Sheng Zhou, Da Li, Yi-Ming Zhou, Ji-Min Cao

**Affiliations:** 1 Department of Physiology, Medical College, Dalian University, Dalian, 116622, China; 2 Department of Physiology, China Medical University, Shenyang, 110001, China; 3 Section of Cell Signaling, Okazaki Institute for Integrative Bioscience, National Institutes of Natural Sciences, Okazaki, 444-8787, Japan; 4 Department of Physiology and Pathophysiology, Institute of Basic Medical Sciences, Chinese Academy of Medical Sciences, School of Basic Medicine Peking Union Medical College, Beijing, 100005, China

**Keywords:** The skin, Antioxidant defense, Xenobiotic, Lipid homeostasis, Sedentary lifestyle, Metabolic syndrome, Acanthosis nigricans, Acne

## Abstract

The body’s total antioxidant capacity represents a sum of the antioxidant capacity of various tissues/organs. A decrease in the body’s antioxidant capacity may induce oxidative stress and subsequent metabolic syndrome, a clustering of risk factors for type 2 diabetes and cardiovascular disease. The skin, the largest organ of the body, is one of the major components of the body’s total antioxidant defense system, primarily through its xenobiotic/drug biotransformation system, reactive oxygen species-scavenging system, and sweat glands- and sebaceous glands-mediated excretion system. Notably, unlike other contributors, the skin contribution is variable, depending on lifestyles and ambient temperature or seasonal variations. Emerging evidence suggests that decreased skin’s antioxidant and excretory functions (e.g., due to sedentary lifestyles and low ambient temperature) may increase the risk for metabolic syndrome. This review focuses on the relationship between the variability of skin-mediated detoxification and elimination of exogenous and endogenous toxic substances and the development of metabolic syndrome. The potential role of sebum secretion in lipid and cholesterol homeostasis and its impact on metabolic syndrome, and the association between skin disorders (acanthosis nigricans, acne, and burn) and metabolic syndrome are also discussed.

## Introduction

The metabolic syndrome (MetS), which is characterized by obesity, insulin resistance, dyslipidemia, and hypertension, is thought to be a driver of the modern-day epidemics of type 2 diabetes and cardiovascular disease [1,2]. Over the past few decades, there has been an alarming increase in the prevalence of MetS. Approximately one third of the adult population in developed countries can be categorized as having MetS by different definitions [3]. Most evidently, the rise of the epidemic of obesity, a major component of MetS, seemed to begin almost concurrently in most high-income countries in the 1970s and 1980s, which is thought to result from changing global food system and increasingly sedentary lifestyles, especially the former [4]. However, the exact cause remains under investigation.

Oxidative stress, which is thought to play a central pathogenic role in the pathogenesis of MetS, is a condition of oxidant/antioxidant imbalance in which the net amount of reactive oxygen species (ROS) exceeds the antioxidant capacity of the body [1,5]. Excessive ROS can react with cellular macromolecules and cause lipid peroxidation, protein oxidation, and oxidative DNA damage [1]. One of the major sources of ROS is xenobiotics which are exogenous chemicals, including drugs, environmental pollutants, cosmetics, and even components of the diet [6-8]. Notably, over the past few decades, excessive xenobiotic exposure has occurred in the general population, for example due to food additives and synthetic-nutrient supplements [9]. In this case, there is the possibility that xenobiotics might be involved in increased prevalence of MetS and related diseases [2].

The skin, which is the body's largest organ, plays a role in the metabolism and elimination of xenobiotics, endogenous bioactive substances, lipids, and cholesterol [10-15]. This review focuses on the relationship between the variability of skin-mediated detoxification and elimination of exogenous and endogenous toxic substances and the development of MetS. The potential role of sebum secretion in lipid and cholesterol homeostasis and its impact on MetS, and the association between skin disorders (acanthosis nigricans, acne, and burn) and MetS are also discussed.

## Xenobiotics, oxidative stress and metabolic syndrome

Xenobiotics, which are encountered by humans on a daily basis, undergo metabolism and detoxification to produce numerous metabolites, some of which have the potential to cause toxic effects [8]. Xenobiotics are degraded or biotransformed by two enzyme systems called phase I and II, and eliminated from the body through urine and sweat and other excretory pathways. The following evidence suggests a possible involvement of xenobiotics in the pathogenesis and prevalence of MetS.

1) Xenobiotic metabolism in the body generates ROS and high exposure to xenobiotics can lead to oxidative stress [6,7,16].

2) The degradation of many xenobiotics involves methylation, a methyl-consuming reaction [2]. Therefore, high xenobiotic exposure may disturb the methylation of endogenous substrates due to competition for labile methyl groups. For example, excess nicotinamide (a form of niacin) can inhibit methylation-mediated degradation/inactivation of catecholamines, resulting in an increase in the levels of circulating norepinephrine [17], a phenomenon commonly seen in MetS [18], which provides the first evidence that methyl-consuming xenobiotics may contribute to increased circulating norepinephrine. In animal studies, arsenic, a common environmental methyl-consuming toxin that increases the risk of MetS [19], is found to cause global DNA hypomethylation [20,21].

3) There has been increasing evidence that numerous xenobiotics, such as heavy metals [7,19], organic pollutants [22-24], and long-term medications (e.g., atypical antipsychotic [25], anti-bipolar disorder [26], and anti-cancer medications [27]), may play a causal role in MetS.

4) Lipid metabolism also increases the demand for methyl groups (due to the synthesis of phosphatidylcholine from phosphatidylethanolamine) and methyl deficiency causes hepatic steatosis and subsequent plasma dyslipidemia [28]. Thus, high fat intake may have synergy with xenobiotics in the development of MetS.

5) Strictly speaking, synthetic vitamins also belong to the xenobiotic group, because excessive amounts of vitamins, such as niacin (nicotinamide and nicotinic acid) [29], vitamin D [30], vitamin E, and vitamin K [31], are degraded by xenobiotic/drug-metabolizing enzymes. Our ecological evidence has suggested a strong positive lag-correlation between the prevalence of obesity and diabetes and the consumption of B-vitamins (niacin, thiamin, and riboflavin) in the U.S., primarily due to mandatory food fortification [9]. Moreover, among the B-vitamins, niacin has been known to cause hepatic toxicity [32], insulin resistance [33,34], and oxidative stress [29,35].

6) The metabolism of xenobiotics and ROS involves numerous enzymes. The polymorphisms in the genes of xenobiotic/drug-metabolizing enzymes and ROS-scavenging enzymes are expected to be involved in susceptibility to xenobiotic exposures and MetS. Indeed, evidence has shown that the gene polymorphisms of many of the enzymes, such as *N*-acetyltransferase 2 [36], superoxide dismutases [37], peroxiredoxins [38], glutathione *S*-transferase [39], and NAD(P)H oxidase [40], may play a crucial role in determining genetic susceptibility to metabolic disorders. Moreover, current research also shows correlations between polymorphisms in xenobiotic/drug-metabolizing enzymes and cancer susceptibility [41-43].

7) Dysfunctions of the organs that are responsible for the biotransformation and excretion of xenobiotics, such as the liver and kidney [44,45], may increase the risk for MetS.

**Figure 1:**
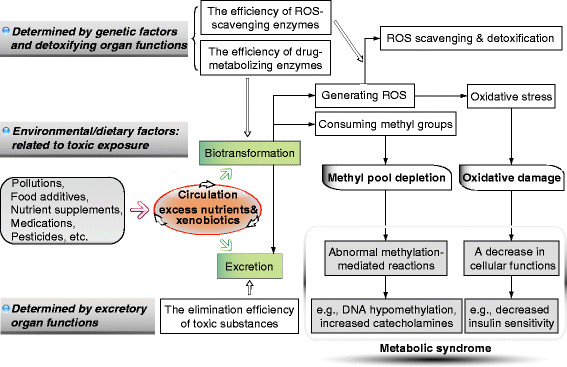
**Possible link among environmental/dietary factors, genetic factors, oxidative stress, and aberrant methylation profile in MetS.** Increased xenobiotic and synthetic-nutrient exposure may be the primary cause of MetS (See text and Ref. [2] for further details). ROS, reactive oxygen species.

Recently, we hypothesize that MetS may be a consequence of chronic xenobiotic poisoning [2], which may involve a mechanism of xenobiotic-induced systemic tissue damage and subsequent decrease in cellular response to physiological signals (e.g., insensitive to insulin [46,47]), and methyl depletion and subsequent disturbance in numerous methylation-mediated reactions in the body (e.g., inhibition of catecholamine degradation [17]). All of the above evidence suggests that environmental/dietary factors and genetic factors in MetS may be, to some extent, a reflection of xenobiotic exposure and the efficiency of the body’s xenobiotic-biotransforming/eliminating system and ROS-scavenging system (Figure 1). Therefore, it seems conceivable that any tissues/organs that contribute to the body’s total antioxidant capacity might play some role in the development of MetS.

## The skin’s antioxidant and excretory systems

The skin provides both a mechanical and a chemical barrier between the body and surrounding environment. The chemical barrier is mediated by the skin’s xenobiotic biotransformation system, ROS-scavenging system, and excretory system, all of which contribute to the body’s total antioxidant capacity. The following sections will discuss the skin’s function and its role in MetS.

### Xenobiotic-metabolizing and ROS-scavenging systems of the skin

Xenobiotic-metabolizing phase I and II enzymes are expressed differently in various tissues, determining the antioxidant capacity of a given tissue or organ. The skin expresses all known phase I and II enzymes, such as cytochrome P450 enzymes, flavin-dependent monooxygenase, monoamine oxidase, alcohol dehydrogenase, aldehyde dehydrogenase, NADP(H): quinone oxidoreductase, glutathione *S*-transferase, and catechol-*O*-methyhransferase [14,48]. The xenobiotic-metabolizing enzymes are induced in response to xenobiotic exposure [49,50]. Moreover, endogenous bioactive and toxic substances, such as catecholamines and steroids [48-51], are also substrates of phase I and II enzymes. Moreover, the skin is also equipped with an antioxidant system. For example, the skin expresses superoxide dismutase, catalase, and glutathione peroxidase, which can remove ROS [52].

The expression of both xenobiotic/drug-metabolizing and ROS-scavenging enzymes suggests that the skin may contribute to the body’s total antioxidant defense. To test this hypothesis, our previous study investigated the role of the skin in the degradation of nicotinamide, a known insulin resistance inducer [34], by using a rat burn model, and found that rats underwent a 40% total body surface area burn injury exhibited a significantly higher baseline plasma *N*^1^-methylnicotinamide (the toxic intermediate metabolite of nicotinamide) levels than sham-treated rats [29]. A nicotinamide (100 mg/kg body weight, i.p.) plus glucose (2 g/kg body weight, i.p.) loading test further revealed that the tolerance of burned rats to nicotinamide significantly decreased, which was characterized by high levels of plasma nicotinamide and *N*^1^-methylnicotinamide associated with high levels of plasma H_2_O_2_ (a form of ROS) and insulin after co-administration of nicotinamide and glucose (unpublished data). These findings suggest that the skin may be a major component of the body’s total antioxidant defense.

### Sweat-mediated elimination of toxic substances

The skin also acts as an excretory organ. It is estimated that 3 to 4 million eccrine sweat glands which together roughly weigh the same as one kidney (i.e., 100 g) are distributed over almost the entire human body surface. An individual can perspire as much as several liters per hour and approximately 10 liters per day [11]. Water-soluble exogenous and endogenous toxic/bioactive substances, such as metals [11], drugs [10], cytokines [53], and steroids [54], can be eliminated in the sweat. Genuis *et al.*[55] analyzed for approximately 120 various compounds, including toxic elements, and found that many toxic elements appeared to be preferentially excreted through sweat. It is worth noting that some xenobiotics that are rarely excreted in the urine without being metabolized, but can be excreted in the sweat. For example, excess nicotinamide cannot be eliminated through urine because of its reabsorption by the renal tubules, but it can be effectively excreted by the sweat gland [29].

Xenobiotic metabolism may produce toxic intermediate products and ROS [6-8,16,29]. Therefore, sweating-induced elimination of xenobiotics is expected to reduce the production of toxic intermediate products and thus prevent oxidative stress. Indeed, our previous study found that the levels of nicotinamide but not its toxic intermediate metabolite *N*^1^-methylnicotinamide in the sweat significantly increased after nicotinamide loading [29]. Masuda *et al*. have also observed that sauna, which increases the skin temperature and induces sweating, can protect against oxidative stress [56]. Moreover, sauna has been found to alleviate symptoms of intoxication [57,58] and improve lifestyle-related diseases [59-61]. The beneficial effect of saunas is thought to be related sweat-mediated elimination of toxic substances from the body [61]. Although not proved, increased skin temperature-induced changes in the activity of xenobiotic- and ROS-metabolizing enzymes may also contribute to the beneficial effect of sauna. Thus, it appears that sweating might be an important antioxidant mechanism, especially for individuals who have a genetic enzymatic defect in dealing with xenobiotics and ROS.

### Sebum-mediated elimination of excess lipids and cholesterol

It is known that there are two major pathways for the elimination of water-soluble compounds (including excessive nutrients) from the circulation: urine and sweat. As for the elimination of excessive circulating lipids and fat-soluble substances, sebum secretion may be an important pathway, though this factor has received little attention in the investigation of lipid homeostasis.

Sebaceous gland, which produces sebum, is found throughout the human body except on the palms of the hands and soles of the feet. Sebum is composed of triglycerides, fatty acids, cholesterol, squalene, and wax esters. The major component of human sebum is triglycerides and fatty acids (57%), which is much higher than that of other species, such as rodents and rabbit (their triglycerides and free fatty acids <10%) [15]. Studies have shown that the production of sebum is linked to diet, for example, caloric deprivation decreases the production of sebum [62,63], whereas a high fat diet significantly increases it [64]. Since an increase in energy intake mainly increases the excretion of triglycerides and cholesterol and its esters in sebum, but not of squalene [62,63], it appears that the major function of sebum secretion may be to eliminate excessive lipids and cholesterol from the body, and thus play a role in maintaining lipid and cholesterol homeostasis. This notion is supported by the observation that inhibition of sebum secretion by isotretinoin significantly increases plasma triglyceride and cholesterol levels [65,66].

### Factors affecting skin antioxidant and excretion efficiencies

The skin function is affected by external factors, such as lifestyles and working conditions [67]. Among known factors, temperature may be probably the most important. The optimum temperature for human enzymes, including skin biotransformation enzymes [12,68], is about 37 °C. The activity of enzymes in internal organs is rather stable, because the core temperature of the body is maintained at a constant level close to 37 °C; but the activity of skin enzymes changes with the skin temperature, which is considerably affected by ambient temperature [69]. Moreover, the activity of sweat glands is also conditional. During heat exposure, increases in body temperature trigger cutaneous vasodilation and sweating. With hyperthermia in humans, blood flow to the skin can increase from approximately 250 mL/min in thermoneutral environments to as much as 6 to 8 L/min or 60% of the cardiac output [70]. A heat exposure-induced increase in blood flow to the skin and the skin temperature could, in theory, increase: 1) the activity of skin enzymes; 2) the probability of enzymes catching toxic substances in the circulation; 3) sweat-mediated elimination of toxic substances, because water-soluble toxic substances can be excreted in sweat [10,11,53,54]; and 4) sebum-mediated elimination of circulating lipids and cholesterol, because sebum secretion is temperature-dependent [71-73]. All of these changes during heat exposure strengthen the body’s antioxidant defense and increase the excretion of circulating lipids and cholesterol. On the contrary, upon exposure to cold environments, blood flow to the skin decreases via cutaneous vasoconstriction [70], which results in a decrease in the functions of the skin. Obviously, changes in skin function might lead to changes in the body's total antioxidant capacity.

## Sedentary lifestyles and skin’s antioxidant efficiency

A sedentary lifestyle is associated with an increased risk of MetS [74,75], which is usually attributed to decreased energy expenditure. Indeed, moderate-to-vigorous physical activity, which increases energy expenditure, may produce beneficial effects [76-78]. However, recently, Thorp and colleagues [74], after having reviewed forty-eight studies published between 1996 and January 2011 on sedentary behaviors and subsequent health outcomes in adults, concluded that the effect of sedentary behavior on health outcomes may be independent of physical activity. Moreover, Sisson *et al.*[79] examined leisure time sedentary behavior and usual occupational/domestic activity and their relationship with MetS and individual cardiovascular disease risk factors, and also found that usual occupational/domestic activity was not strongly associated with MetS or CVD risk factors, although their data also showed an association between sedentary behavior and MetS. It seems that the effect of sedentary lifestyles on MetS may be not just a matter of decreased energy expenditure.

The body usually does not sweat at room temperature (i.e., living a sedentary lifestyle), which may reduce sweat-mediated elimination of toxic substances and excess nutrients from the body. In contrast, sweating-inducing factors, such as sauna and exercise, can facilitate the elimination, as discussed above. Unfortunately, in most studies on sedentary lifestyle and exercise and their relationship with MetS and related diseases, the accompanying changes in skin contribution to the body’s antioxidant capacity are usually neglected.

It might be worth noting that exercise might be a double-edged sword, for it also increases the generation of ROS and subsequent risk of oxidative stress [80]. Moreover, not everyone is healthy enough for exercise that induces sweating. In contrast, sweating induced by exposure to a hot environment (natural sweating) could not only effectively eliminate toxic substances from the body, but it also avoid the generation of ROS through muscle activity. Theoretically, natural sweating might be more effective and practical than exercise-induced sweating in the prevention and treatment of MetS.

In addition, Pearson and Biddle [75], after having reviewed fifty-three relevant studies, concluded that sedentary behavior is clearly associated with unhealthy diet including lower fruit and vegetable consumption and higher consumption of snacks and fast foods. Because snacks and fast foods, which are used as a fortification vehicle [81], have much higher concentrations of synthetic vitamins than other food items (e.g., the amount of niacin in ready-to-eat cereals is 76 mg/pound in 1974-2000 according to the fortification recommendations [81]), high consumption of these foods may lead to an excessive synthetic-vitamin intake, whereas sedentary lifestyle may decrease skin-mediated elimination of toxic substances and excess nutrients, as discussed above. The combination of these two factors might play a major role in the development of MetS.

## The skin function and obesity

**Figure 2:**
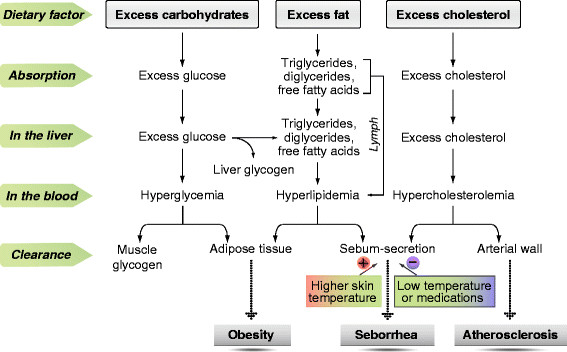
**Relationships among excess energy intake, skin function and health outcomes.** +, stimulation; –, inhibition.

Obesity is the result of a chronic excess energy intake. As shown in Figure 2, excess dietary carbohydrates can be converted either into liver and muscle glycogen [82] or into fat in adipose tissue [83], while excess dietary fat, besides being stored as body fat, can also be eliminated in the form of sebum. In the circumstances of chronic excess energy intake and inhibition of sebum secretion, excess lipids are only stored as adipose tissue, whereas excess cholesterol can accumulate in the arterial wall, which has long been recognized [84]. As a result, obesity and atherosclerosis may occur. This notion is supported not only by the observations that there is usually low sebum secretion [71-73] but high blood lipid levels [85-88] and weight gain [89,90] in winter, but also by the findings that medication-induced inhibition of sebum secretion increases the levels of circulating lipids and cholesterol, and, consequently, the risk of dyslipidemia and MetS [65,66,91].

**Figure 3:**
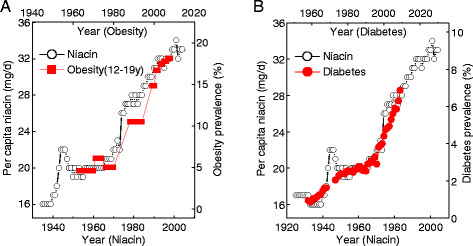
**Trends in U.S. per capita niacin consumption and the prevalence of obesity and diabetes.** The prevalence of obesity (A) and diabetes (B) increased in parallel with the increase in U.S. per capita niacin consumption with a lag of 10 years and 26 years, respectively (see Ref. [9,35] for detail). The sharp increase in niacin consumption in 1940s and 1974 is due to the implementation of mandatory grain fortification and the update of fortification standards, respectively. The data on the prevalence of obesity and diabetes are from the U.S. Centers for Disease Control and Prevention (http://www.cdc.gov/nchs/data/hestat/obesity_child_07_08/obesity_child_07_08.htm; and http://www.cdc.gov/diabetes/statistics/slides/long_term_trends.pdf. Accessed March 24, 2012). The data on U.S. per capita consumption of niacin is from Economic Research Service: Nutrient Availability Spreadsheets, (http://www.ers.usda.gov/data/foodconsumption/NutrientAvailIndex.htm. Accessed March 24, 2012).

**Figure 4:**
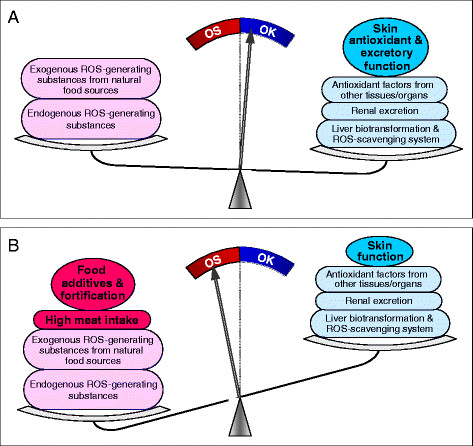
**Factors affecting the balance between ROS production and scavenging.** A, In traditional lifestyles, ROS are derived from the metabolism of endogenous and exogenous (i.e., natural dietary) substances. The skin, especially its sweat glands, may play an important antioxidant role. B, In modern lifestyles, dietary xenobiotics have significantly increased, while the skin functions, especially sweat-mediated excretion, is decreased due to sedentary lifestyles. As a result, an imbalance between ROS production and the body’s antioxidant defense system takes place. OK, antioxidant defense capacity > ROS production; OS, oxidative stress.

Studies have revealed that: (1) the driver for the global obesity epidemic may be in the food system [4], (2) food insecurity leads to obesity [92,93], (3) there is a correlation between the epidemic of obesity and diabetes and increased exposure to synthetic B-vitamins (niacin, thiamin, and riboflavin) due to food fortification (Figure 3), (4) obese and overweight persons have uncontrollable eating [94,95], and (5) evidence suggests that oxidative stress caused dysregulated production of inflammation-related adipocytokines (fat-derived hormones) [96-98]. We therefore suspected that xenobiotics might be related to the etiology of uncontrollable eating in obesity. Our previous work tested this hypothesis by conducting oral glucose tolerance tests with or without the presence of nicotinamide in the same healthy subjects, and, as expected, found that co-loading of glucose and nicotinamide triggered a hypoglycemic reaction (i.e. low blood glucose levels with hunger feeling) in the later phase of the loading test (3 h) due to increased ROS generation and insulin resistance occurred in the earlier phase [35]. This finding provides evidence, for the first time, that dietary xenobiotics may play a primary causal role in uncontrolled eating. Sweating can excrete toxic substances [10,11,55], including excess nicotinamide [29]. Thus, sweating-mediated toxin elimination is expected to prevent eating disorders. In agreement with this notion, Biro *et al*. found that sauna could effectively reduce not only body weight and body fat but also the abnormal eating behavior in obesity [59]. All of the above evidence suggests that the skin antioxidant and excretory functions may play a role in obesity.

## The skin function and seasonal variations of metabolic syndrome

Metabolic disorders share two common features: 1) regional differences in their prevalence and 2) seasonal variations in their symptoms and signs. The prevalence of MetS and related diseases in high-latitude regions is higher than that in low-latitude regions; for example, the prevalence of hypertension, obesity and cardiovascular disease is higher in North China than in South China [99-101]. Blood pressure also shows seasonal fluctuations (higher in cold season) [85,102]. Kamezaki *et al*. found that the prevalence rates of MetS are higher in winter than in summer [85]. One of the most compelling factors contributing to the seasonal and regional variations may be temperature. Indeed, two recent studies after three-year observations have found a negative correlation between blood pressure and outside temperature [103,104]. Kimura and colleague*s* found that a 1 °C decrease in the mean outdoor temperature was associated with rises of 0.43 mmHg in systolic blood pressure and 0.29 mmHg in diastolic blood pressure [103]. Hozawa *et al*. reported that when the outside temperature was ≥ 10 °C, 1 °C increment of outside temperature corresponded to 0.40 and 0.28 mmHg decrease of systolic blood pressure and diastolic blood pressure [104]. Thus, it is possible that the skin may mediate the association between blood pressure fluctuations and ambient temperature, because the skin is more vulnerable to environmental temperature than other organs in the body. Although there currently is no systematic study on this issue, several lines of evidence suggest that the seasonal variation of MetS may involve seasonal fluctuations in skin-mediated elimination, excretion and biotransformation of toxic substances and excess nutrients, such as lipids, catecholamines, and niacin.

Numerous studies have shown that there is a substantial increase in blood cholesterol level in winter [85-88], while the data on the seasonal variation of serum triglycerides appear to be inconsistent [85,88]. Low ambient temperature decreases the elimination of triglycerides and cholesterol due to a reduction in sebum secretion [71-73], which is expected to raise the level of both triglycerides and cholesterol in the blood. However, excess triglycerides can be stored as fat in adipose tissue (indeed, body mass may increase in winter [89,90]), while excess cholesterol would likely be left in the blood streams in decreased sebum-secretion condition. As a result, the serum cholesterol level is elevated in winter. This interpretation is supported by the observation that inhibition of sebum secretion by isotretinoin may lead to an increase in both serum triglyceride and cholesterol levels [65,66,91]. Unlike the effect of cold exposure, which may be regional and dependent upon clothing condition, the effect of isotretinoin should be an overall sustained inhibition of the sebaceous glands in the body. This may explain the increase in serum triglyceride level during isotretinoin treatment.

Catecholamines, which mediate the cardiovascular effects of the adrenergic nervous system, are degraded/inactivated by monoamine oxidase and catechol-*O*-methyhransferase [105]. An increase in the degradation of catecholamines generates more end metabolites (i.e., homovanillic acid and vanillylmandelic acid), while a decrease in the degradation might result in an increase in the levels of circulating catecholamines [17]. The skin expresses monoamine oxidase and catechol-*O*-methyhransferase [48], and thus might play a role in the inactivation of circulating catecholamines. Studies have shown that the blood concentrations of the end metabolites of catecholamines are lower in winter than in summer [106], whereas the levels of plasma norepinephrine and epinephrine are higher in winter than in summer [107]. These observations suggest a decrease in the degradation of catecholamines in winter. It seems that there is a negative relationship between the degradation rate of catecholamines and the seasonal fluctuations of blood pressure. Given that 1) seasonal changes may change the skin temperature and subsequent the activities of cutaneous catecholamine-degrading enzymes, but do not change the core temperature and the enzyme activity in internal organs; and 2) water-soluble free amino acids and neurotransmitters can be excreted in sweat [11], it appears that the seasonal variation in circulating catecholamines might be related to the seasonal variation in skin functions.

Niacin is precursor of NAD and NADP, which are coenzymes in numerous essential redox reactions in cellular metabolism. Niacin deficiency causes pellagra, while excessive nicotinamide may increase the risk for MetS, for it induces oxidative stress and insulin resistance [29,33-35] and disturb the degradation of catecholamines [17]. Pellagra occurs mostly in the summer months (i.e., a season facilitating sweating) in rural poor people who consume a niacin-poor diet [108], while MetS is worse in winter (i.e., a season inhibiting sweating) [85], and commonly occurs in people who consume a niacin-fortified diet [9]. Since niacin can be excreted in the sweat [29], these phenomena may involve sweating-mediated niacin elimination, but further studies needed to confirm this.

## Skin diseases and metabolic syndrome

Numerous studies have shown that a variety of skin disorders are frequently associated with metabolic disorders. Based on available evidence, it seems that the associations might involve skin antioxidant and excretory functions.

### Acanthosis nigricans

Acanthosis nigricans, a hyperplastic skin lesion, is associated with insulin resistance, obesity, MetS, and type 2 diabetes [109,110]. The following evidence suggests that acanthosis nigricans might be linked to skin-mediated xenobiotic detoxification:

· Long-term exposure to xenobiotics, such as niacin, glucocorticoids, and oral contraceptives, increases the risk for both acanthosis nigricans [109] and MetS [111].

· There has been a significant increase in xenobiotic exposure in general population due to food additives and especially mandatory implementation of synthetic vitamin fortification [9].

· The prevalence of obesity, which is closely associated with acanthosis nigricans [109], is positively correlated with niacin consumption [9,35].

· Physical activity, which can increase the sweaty excretion of xenobiotics, reduces the risk of acanthosis nigricans [112].

Since xenobiotics induce xenobiotic-metabolizing enzymes [49,50] and cell proliferation [113,114], it appears that increased cell proliferation in acanthosis nigricans might be a compensatory mechanism in response to chronic high xenobiotic exposure, but further studies are needed to test this hypothesis.

### Acne

Acne, a common skin condition, is closely related to increased sebum production [115]. As mentioned above, increased sebum secretion may be helpful to remove excess lipids and cholesterol from the body. Therefore, in theory, inhibition of sebum secretion would increase the accumulation of lipids and cholesterol in the body. In fact, numerous studies have found that long-term medication-induced inhibition of sebum secretion can lead to significant increases in the levels of lipids and cholesterol in the circulation, and consequently increase the risk of MetS [91] and atherosclerosis [65,66]. Given that sebum secretion is positively related to caloric intake [62,63], it seems that increased sebum secretion, although increasing the risk of acne, may be a protective mechanism in response to excess energy intake. Indeed, in a retrospective follow-up study of 11,232 men who attended Glasgow University between 1948 and 1968 and whose mortality was traced into 2004, the authors found that participants who reported having acne during adolescence had a significantly lower risk of death from coronary heart disease [116]. The analysis of the available literature suggests that the best solution for solving the acne problems may be to reduce appetite and total energy intake, rather than to inhibit sebum secretion with medications.

### Burns

Severe burns covering >40% of the total body surface area lead to profound metabolic changes. Insulin resistance is one of the most prominent post-burn metabolic abnormalities. Burn-induced insulin resistance, unlike other trauma-induced temporary insulin resistance, is a long-lasting phenomenon, and it still persists after burn wounds have already healed [117]. Obviously, severe skin burns cause a permanent decrease in or loss of the biotransformation, detoxification, antioxidant, and excretory functions in the burned area, which may cause a permanent decrease in the body’s total antioxidant capacity due to decreased skin contribution. If this were the case, it is expected that there would still be an accumulation of toxic and bioactive substances in the circulation after burn injuries have healed. Indeed, a recently published study has found that healed severe burns are still associated with an elevation in circulating cortisol, catecholamines, and cytokines, all of which are insulin resistance-inducing factors [118]. Therefore, it appears that post-burn metabolic disorders might be due to a permanent decrease in the skin-contribution to the body’s antioxidant capacity.

## Conclusion and perspectives

The imbalance between ROS generation and the body total antioxidant defense system in MetS may be a consequence of the combination of excessive xenobiotic exposure (including fortification-induced high synthetic-vitamin exposure) and decreased detoxification/elimination of xenobiotics due to lifestyle and genetic factors. The skin’s antioxidant and excretory function may be one of the major components of the body’s antioxidant defense system and play an important role in anti-MetS (Figure 4).

The physiological functions of skin might be probably more complex than expected. In this review we focused solely on the possible relationship between the skin detoxification and excretory functions and MetS. The skin, without doubt, has some other important functions, for example, it is involved in the metabolism of many endogenous bioactive substances and some vitamins. Although the basic functions of skin have been well documented, the role of skin in systemic metabolic disorders is far from clear. Therefore, further studies are required for deep understanding of the role of the skin in the development of MetS.

## Competing interests

The authors declare that they have no competing interests.

## Authors' contributions

SSZ conceived and drafted the manuscript and the figures, DL participated in the preparation of the figures, DL and YMZ contributed to the acquisition of data and revised the manuscript, JMC reviewed the manuscript critically. All authors read and approved the final manuscript.
